# A comparison of quality of life between patients treated with different dialysis modalities in Taiwan

**DOI:** 10.1371/journal.pone.0227297

**Published:** 2020-01-06

**Authors:** Chu-Chun Hsu, Chiu-Ching Huang, Yue-Cune Chang, Jin-Shuen Chen, Wen-Chen Tsai, Kwua-Yun Wang

**Affiliations:** 1 Department of Nursing, Tri-Service General Hospital, Taipei City, Taiwan (R.O.C.); 2 School of Nursing, National Defense Medical Center, Taipei City, Taiwan (R.O.C.); 3 Department of Internal Medicine, Division of Nephrology and Kidney Institute, China Medical University and Hospitals, Taichung City, Taiwan (R.O.C.); 4 College of Medicine, China Medical University, Taichung City, Taiwan (R.O.C.); 5 Department of Mathematics, Tamkang University, New Taipei City, Taiwan (R.O.C.); 6 Department of Internal Medicine, Division of Nephrology, Tri-Service General Hospital, Taipei City, Taiwan (R.O.C.); 7 National Defense Medical Center, Taipei City, Taiwan (R.O.C.); 8 Department of Health Services Administration, China Medical University, Taichung City, Taiwan (R.O.C.); 9 Department of Nursing, Taipei Veterans General Hospital, Taipei city, Taiwan (R.O.C.); University of Wisconsin, UNITED STATES

## Abstract

**Purpose:**

This study compared the quality of life (QOL) of hemodialysis (HD) and peritoneal dialysis (PD) patients in Taiwan.

**Methods:**

This cross-sectional study recruited end-stage renal disease patients from 34 Taiwanese hospitals or clinics. Patient characteristics, diagnoses, and laboratory data were extracted from charts. The Chinese version of the Quality of Life Index–Dialysis version (QLI-D) was used. Multiple linear regression analysis showed the effects of dialysis modality on QOL. P<0.05 indicated statistical significance.

**Results:**

In total, 600 HD and 387 PD patients were included. The mean health and functioning, social and economic, psychological/spiritual, and family subscale scores and total QOL scores were significantly lower in HD patients than PD patients. After adjusting for region, hospital level, age, education level, marital status, and Karnofsky Performance Scale, the total QOL was 2.81 points higher for PD patients than for HD patients visiting medical centers (p<0.001). The total QOL was 2.53 points lower in PD patients than in HD patients for those visiting clinics.

**Conclusion:**

Compared to HD patients, PD patients had better QOL in Taiwanese medical centers. The current survey improves our understanding of the QOL of patients undergoing different dialysis modalities in Taiwan.

## Introduction

Patients with end-stage renal disease (ESRD) should undergo renal replacement therapy (RRT). The choice of therapy is between transplantation and dialysis; the latter can be further subdivided into hemodialysis (HD) and peritoneal dialysis (PD). Dialysis is the most prevalent ESRD treatment in Taiwan. HD is typically performed three times a week using a dialysis machine in an outpatient facility under the supervision of nurses. PD patients receive training by professional nursing staff and typically self-administer dialysis at home autonomously or with the help of a caregiver. PD is performed either by the manual exchange of dialysis fluid 4 times a day (continuous ambulatory peritoneal dialysis, CAPD) or by using a machine that automatically fills and drains the peritoneum while the patient is asleep (automated peritoneal dialysis, APD). These dialysis modalities profoundly influence patients’ lives.

In Taiwan, HD and PD patients were observed to have similar long-term survival [1]. Thus, in the absence of medical contraindications, the decision as to which dialysis modality should be used becomes a matter of personal choice. Such a decision requires thoughtful consideration of the value a patient places on the potential gains or losses with regard to quality of life (QOL) associated with each treatment.

QOL is an important indicator of the effectiveness of treatment. Ferrans and Powers [2] mention that QOL is related to one’s satisfaction with the perceived important life events for individuals. They proposed a QOL index (QLI) to evaluate the QOL of healthy individuals as well as the QOL of those with an illness. The instrument consists of two sections: one measures satisfaction in various domains of life and the other measures the importance of the domain to the subject. Both sections have 36 items and four subscales: health and functioning, social and economic aspects, psychological and spiritual status, and family and relationships. The QLI has been widely used and validated as a QOL assessment tool for patients undergoing dialysis [3,4].

Many studies on QOL performed with HD and PD patients around the world have produced conflicting findings. Some studies have shown that the QOL of PD patients is higher than that of HD patients [5–8]. Some studies have shown that QOL is similar between HD and PD patients [9,10]. One study showed that the QOL of HD patients is higher than that of PD patients [11]. However, it is widely accepted that QOL in these patient groups is affected by various factors pertaining to dialysis. In Taiwan, the findings have not been entirely consistent [12,13].

QOL is affected by various factors pertaining to dialysis. The QOL of dialysis patients is affected by demographic variables (age, sex, education level, marital status, and employment status), functional status, duration of dialysis, Charlson comorbidity index (CCI), diabetes as a primary cause, albumin levels, hemoglobin levels, and emotional status (anxiety and depression) [4,5,8,11–13]. Hence, regional and national disparities exist in the study of QOL. Notably, most Taiwanese HD patients receive treatment at local dialysis clinics, and PD patients return to the hospital for treatment. Thus, studies of QOL should consider local- and hospital-level factors.

Randomized controlled trials are considered the gold standard to estimate the effects of treatments and interventions on outcomes. However, because HD and PD differ profoundly, randomized comparisons have proven impossible [14]. Historically, researchers have relied on the use of regression adjustment to account for differences in measured baseline characteristics between HD and PD patients.

Taiwan is a highly endemic region for ESRD [15], and dialysis therapy is still the treatment of choice for most patients. However, a national survey on dialysis patients’ QOL remains lacking. Previous QOL studies assessing dialysis patients in Taiwan were local region-based and hospital-based studies, and the findings were not entirely consistent. Because both modalities continue to evolve substantially from each year, up-to-date QOL comparisons may help inform the choice between modalities for patients and physicians when considering dialysis therapy. Hence, the aim of the study was to compare QOL between patients on HD and those on PD in Taiwan.

## Materials and methods

### Participants

This prospective cross-sectional study was conducted between March 2010 and March 2011. The number and distribution of HD and PD patients in Taiwan in 2009 were retrieved from the website of the Health Promotion Administration. Based on the regions of residence (northern, central, southern, or eastern) and hospital levels (medical center, regional hospital, district hospital, or clinic), patients were recruited from the northern, central, southern, and eastern regions using an equal ratio principle. The inclusion criteria were age >20 years, ability to communicate in Mandarin or Taiwanese, and receiving dialysis for more than 3 months. Patients with cognitive impairment or psychiatric disease were excluded. This study was approved by the institutional review board of Taipei Veterans General Hospital (no: 201001026IC).

### Data collection

After patients’ written informed consent was obtained, data were collected by researchers using self-reported questionnaires and personal health record information. All questionnaires required approximately 30–45 min to be completed. The researchers read the questionnaires to those who were fatigued or had physical difficulty in completing the questionnaires.

### Instruments

The Personal Information Questionnaire and the Chinese version of the Quality of Life Index–Dialysis version (QLI-D) were used to collect data.

The Personal Information Questionnaire included demographic characteristics, disease characteristics, laboratory variables, and the Karnofsky Performance Scale (KPS). The demographic characteristics included region, hospital level, age, sex, education level, employment status, and marital status. The disease characteristics included primary kidney disease, dialysis duration, and the CCI. The laboratory variables included creatinine, hemoglobin, and albumin levels. The KPS is a global indicator of self-sufficiency and functional capacity. It consists of a scale of 11 levels, with scores ranging from 0 to 100; 100 denotes normal function without limitations, whereas 0 signifies death. Individuals with scores ranging from 80 to 100 are capable of normal activity and work, whereas those with scores ranging from 50 to 70 can take care of themselves but cannot work. Patients with scores less than 50 need progressive assistance and care [16].

QLI-D is a disease-specific tool that measures satisfaction and the importance of the determinants of QOL. The scale has 36 items and four subscales: health and functioning, social and economic, psychological/spiritual, and family. The same 36 items are used to measure levels of satisfaction (part 1) and importance (part 2), and scores from parts 1 and 2 are combined. Final scores range from 0 to 30, with higher scores indicating better QOL. The validity of the QLI-D has been documented by Ferrans and Powers [2], with Cronbach’s alpha values ranging from 0.90 to 0.94 [2, 4]. Cronbach’s alpha in the present study was 0.89.

### Statistical analysis

Data analyses were performed using SPSS version 23.0 (IBM, Armonk, New York). The Kolmogorov–Smirnov test of normality was used for data distribution analysis. Normally distributed data and non-Gaussian data are presented as the mean ± SD and median (25th, 75th percentile), respectively. Student’s t-test, the nonparametric Mann–Whitney U test, and the chi-square test were used to examine differences between the two patient groups. Furthermore, univariate linear regression analysis was performed to explore possible prognostic factors or confounding variables affecting QOL. Multiple linear regression analysis was used to examine whether the influence of these prognostic factors on QOL was different between HD and PD patients by adding the interaction terms of dialysis modality and those factors after adjustment for the effects of other confounding variables. Statistical significance was set at p<0.05.

## Results

### Demographic and clinical characteristics

There were 600 HD patients and 387 PD patients. Among the PD patients, 224 patients received continuous ambulatory peritoneal dialysis (CAPD), and 163 patients received APD. Because no significant differences were found between CAPD and APD patients for our study outcomes (S1 Table), we combined CAPD and APD patients into one PD group in all subsequent comparative analyses.

The results for the comparisons of demographic and clinical characteristics between the HD and PD groups are shown in Table 1. Significant differences in almost all demographic characteristics, except for sex and marital status, were noted between the two groups (p = 0.049 and 0.192, respectively). More specifically, 49.6% of PD patients lived in the northern region of Taiwan (vs. 40.3% for the HD group), 58.9% of PD patients visited medical centers, and 41.8% of HD patients visited clinics. The PD patients were significantly younger than the HD patients (p<0.001). The education levels of PD patients were significantly higher than those of HD patients (p<0.001). Notably, 59.9% of PD patients had an education level of senior high school or above (32.3% were college or above), whereas 43.3% of HD patients had an education level of less than or equal to elementary school. When we compared disease characteristics, as shown in Table 1, no significant differences in all primary kidney diseases and CCI, except for dialysis duration, were found between the two groups. However, significant differences in all three laboratory results, namely, the creatinine, hemoglobin and albumin levels, were found (p<0.001, 0.014, and <0.001, respectively).

**Table 1:** Patient demographics, disease characteristics, and relevant laboratory data between HD and PD patients.

Variable	HD (N = 600)	PD (N = 387)	p-value
	N (%)/median (IQR)	N (%)/median (IQR)	
**Demographic characteristics**			
Region			0.005^b^
Northern	242 (40.3)	192 (49.6)	
Central	111 (18.5)	78 (20.2)	
Southern	233 (38.8)	109 (28.2)	
Eastern	14 (2.3)	8 (2.1)	
Hospital levels			<0.001 ^b^
Medical center	53 (8.8)	228 (58.9)	
Regional hospital	145 (24.2)	128 (33.1)	
District hospital	151 (25.2)	26 (6.7)	
Clinic	251 (41.8)	5 (1.3)	
Age	60.8 (53, 70)	53.7 (45, 63)	<0.001^a^
Sex			0.049 ^b^
Female	298 (49.7)	217 (56.1)	
Male	302 (50.3)	170 (43.9)	
Education level			<0.001 ^b^
Elementary school or lower	260 (43.3)	91 (23.5)	
Junior high school	114 (19.0)	64 (16.5)	
Senior high school	144 (24.0)	107 (27.6)	
College or higher	82 (13.7)	125 (32.3)	
Employment status			0.002 ^b^
Unemployed	469 (78.2)	268 (69.3)	
Employed	131 (21.8)	119 (30.7)	
Marital status			0.192 ^b^
Other (single, widowed, divorced)	157 (26.2)	116 (30.0)	
Married/living with someone	443 (73.8)	271 (70.0)	
**Disease characteristics**			
Dialysis duration (years)	6.8 (2.7, 9.5)	3.3 (1.3, 4.2)	<0.001^a^
Primary kidney disease			
Diabetic nephropathy	187 (31.2)	105 (27.1)	0.175 ^b^
Chronic glomerulonephritis	206 (34.3)	146 (37.7)	0.277 ^b^
Hypertensive nephropathy	96 (16.0)	52 (13.4)	0.271 ^b^
Others	180 (30.0)	99 (25.6)	0.132 ^b^
CCI	2.7 (2, 3)	2.6 (2, 3)	0.150 ^a^
**Laboratory data**			
Creatinine (mg/dL)	10.5 (8.7, 11.7)	11.5 (9.1, 13.7)	<0.001^a^
Hemoglobin (mg/dL)	10.6 (9.7, 11.3)	10.3 (9.4, 11.3)	0.014 ^a^
Albumin (g/dL)	3.9 (3.6, 4.2)	3.5 (3.2, 3.9)	<0.001^a^
**Karnofsky Performance Scale**	80 (70, 90)	82.1 (70, 90)	0.051^a^

^a^ Mann–Whitney U test

^b^ chi-square test.

HD, hemodialysis; PD, peritoneal dialysis; IQR, interquartile range.

### Comparison of QLI-D scores between HD and PD patients

The results in Table 2 show that the total QOL of PD patients was significantly higher than that of HD patients after eliminating the possible effects from other confounding variables (p<0.001). Similar results were found in all four subscales (health and functioning, socioeconomic, psychological/spiritual, and family). The lowest scores in both groups were reported in health and functioning. The highest scores in both groups were reported in the family domain.

**Table 2:** Comparison of quality of life index scores between HD and PD patients.

Subscale score (0–30)	HD (n = 600) Mean±SD	PD (n = 387) Mean±SD	p-value^a^
Health and functioning	18.09±4.21	19.25±4.31	<0.001
Socioeconomic	18.61±3.84	19.36±4.17	0.004
Psychological/spiritual	18.70±4.85	19.87±5.16	<0.001
Family	21.77±4.68	22.92±4.69	<0.001
Total quality of life	18.88±3.88	19.94±4.14	<0.001

^a^ Student’s t-test.

HD, hemodialysis; PD, peritoneal dialysis; SD, standard deviation.

We first used simple linear regression to explore the possible effects of confounding variables on QOL. As shown in Table 3, almost all selected factors had significant effects on the total QOL or its four subscales, except for sex and dialysis duration. More specifically, the results regarding dialysis modality in Table 3 were consistent with those in Table 2, indicating that PD patients had significantly higher QOL than HD patients with respect to both the total score and all four subscale scores. Patients who visited medical centers had significantly better QOL than patients who visited all three hospital levels, namely, regional hospitals, district hospitals, and clinics (p<0.001 for all). Younger patients with a higher education level or high Karnofsky performance status also had a significantly better QOL. Accordingly, to compare the QOL between PD and HD patients, we needed to adjust for the effects of those potential confounding variables.

**Table 3:** Univariate linear regression analysis of quality of life scores (n = 987).

	Health and functioning		Socioeconomic		Psychological/spiritual		Family		Quality of life index total score	
	B (SE)	p-value	B (SE)	p-value	B (SE)	p-value	B (SE)	p-value	B (SE)	p-value
**Dialysis modality**										
PD vs. HD	**1.16** (**0.28)**	**<0.001**	**0.75** (**0.26)**	**0.004**	**1.17** (**0.32)**	**<0.001**	**1.15** (**0.31)**	**<0.001**	**1.07** (**0.26)**	**<0.001**
**Region**										
Central vs. northern	**1.46** (**0.37)**	**<0.001**	**1.58** (**0.34)**	**<0.001**	**1.51** (**0.43)**	**0.001**	0.78 (0.41)	0.057	**1.40** (**0.35)**	**<0.001**
Southern vs. northern	0.09 (0.31)	0.779	-0.16 (0.29)	0.564	-0.28 (0.36)	0.434	-0.43 (0.34)	0.205	-0.12 (0.29)	0.676
Eastern vs. northern	-0.51 (0.93)	0.586	0.35 (0.86)	0.681	-1.32 (1.09)	0.224	-0.39 (1.03)	0.705	-0.48 (0.87)	0.582
**Hospital levels**										
Regional hospitals vs. medical centers	**-1.97** (**0.36)**	**<0.001**	**-2.56** (**0.33)**	**<0.001**	**-2.88** (**0.42)**	**<0.001**	**-2.19** (**0.39)**	**<0.001**	**-2.32** (**0.33)**	**<0.001**
District hospitals vs. medical centers	**-1.77** (**0.40)**	**<0.001**	**-1.43** (**0.37)**	**<0.001**	**-2.20** (**0.47)**	**<0.001**	**-1.83** (**0.45)**	**<0.001**	**-1.79** (**0.38)**	**<0.001**
Clinic vs. medical centers	**-1.73** (**0.36)**	**<0.001**	**-1.12** (**0.34)**	**0.001**	**-2.14** (**0.42)**	**<0.001**	**-1.71** (**0.40)**	**<0.001**	**-1.68** (**0.34)**	**<0.001**
**Age**	**-0.06** (**0.01)**	**<0.001**	-0.02 (0.01)	0.056	**-0.04** (**0.01)**	**0.001**	-0.01 (0.01)	0.276	**-0.04** (**0.01)**	**<0.001**
**Sex**										
Male vs. female	-0.28 (0.27)	0.308	-0.33 (0.25)	0.198	-0.33 (0.32)	0.302	-0.25 (0.30)	0.402	-0.29 (0.26)	0.256
**Education level**										
Junior high school vs. elementary school or lower	**1.45** (**0.39)**	**<0.001**	**0.72** (**0.36)**	**0.048**	**0.91** (**0.46)**	**0.046**	0.55 (0.43)	0.202	**1.05** (**0.36)**	**0.004**
Senior high school vs. elementary school or lower	**1.42** (**0.35)**	**<0.001**	**0.94** (**0.33)**	**0.004**	**0.92** (**0.41)**	**0.025**	0.39 (0.39)	0.311	**1.06** (**0.33)**	**0.001**
College or higher vs. elementary school or lower	**2.28** (**0.37)**	**<0.001**	**1.88** (**0.35)**	**<0.001**	**2.07** (**0.43)**	**<0.001**	**1.11** (**0.41)**	**0.007**	**1.96** (**0.35)**	**<0.001**
**Employment status**										
Employed vs. unemployed	**1.67** (**0.31)**	**<0.001**	**1.83** (**0.29)**	**<0.001**	**1.77** (**0.36)**	**<0.001**	0.27 (0.35)	0.429	**1.51** (**0.29)**	**<0.001**
**Marital status**										
Married/living with someone vs. other (single, widowed, divorced)	0.27 (0.31)	0.384	**0.68** (**0.28)**	**0.017**	0.41 (0.36)	0.252	**2.44** (**0.33)**	**<0.001**	**0.73** (**0.29)**	**0.01**
**Dialysis duration (years)**	-0.02 (0.03)	0.415	-0.01 (0.03)	0.767	-0.03 (0.03)	0.433	-0.06 (0.03)	0.069	-0.03 (0.03)	0.339
**Diabetic nephropathy**										
Yes vs. no	**-1.32** (**0.30)**	**<0.001**	**-0.77** (**0.28)**	**0.005**	**-1.11** (**0.35)**	**0.001**	0.03 (0.33)	0.926	**-0.95** (**0.28)**	**0.001**
**CCI**	**-0.80** (**0.17)**	**<0.001**	**-0.51** (**0.16)**	**0.001**	**-0.59** (**0.20)**	**0.002**	-0.21 (0.19)	0.266	**-0.60** (**0.16)**	**<0.001**
**Hemoglobin (mg/dL)**	**0.19** (**0.09)**	**0.041**	0.14 (0.09)	0.116	**0.25** (**0.11)**	**0.021**	0.11 (0.10)	0.302	**0.18** (**0.09)**	**0.039**
**Albumin (g/dL)**	**0.82** (**0.24)**	**0.001**	**0.95** (**0.22)**	**<0.001**	**0.89** (**0.28)**	**0.002**	.047 (0.27)	0.081	**0.81** (**0.23)**	**<0.001**
**Karnofsky Performance Scale**	**0.13** (**0.01)**	**<0.001**	**0.08** (**0.01)**	**<0.001**	**0.11** (**0.01)**	**<0.001**	**0.07** (**0.01)**	**<0.001**	**0.10** (**0.01)**	**<0.001**

Abbreviations: B, unstandardized coefficients; SE, standard error; HD, hemodialysis; PD, peritoneal dialysis; CCI, Charlson comorbidity index.

The results of the multiple linear regression analyses for the total QOL and four subscales are presented in Table 4. After adjusting for the effects of the region, hospital level, age, education level, marital status, and KPS, the total QOL of PD patients who visited medical centers was 2.81 units higher than that of HD patients, and the difference was significant (p<0.001). The total QOL of PD patients who visited clinics was 2.53 (5.34–2.81) units lower than that of HD patients. These different phenomena observed in the two hospital levels reached statistical significance (p = 0.002). The possible reason for this is that 58.9% of PD patients visited medical centers, compared with only 8.8% of HD patients. In contrast, 41.8% of HD patients visited clinics, whereas only 1.3% of PD patients did (Table 1). As shown in Table 4, after adjusting for the effects of dialysis modality, region, hospital level, and other factors presented in the model, patients who were younger, had a higher education level, and had a high Karnofsky performance status had a significantly higher total QOL (all p<0.01).

**Table 4:** Results of multiple linear regression analyses to evaluate the potential impact of prognostic factors on the quality of life (n = 987).

	Health and functioning		Socioeconomic		Psychological/spiritual		Family		Quality of life index total score	
	B (SE)	p-value	B (SE)	p-value	B (SE)	p-value	B (SE)	p-value	B (SE)	p-value
**Dialysis modality**										
PD vs. HD	**1.90** (**0.65)**	**0.003**	**2.08** (**0.62)**	**0.001**	**1.85** (**0.30)**	**0.020**	**2.41** (**0.67)**	**<0.001**	**2.81** (**0.55)**	**<0.001**
**Region**										
Central vs. northern									**0.71** (**0.32)**	**0.027**
Southern vs. northern									0.05 (0.26)	0.862
Eastern vs. northern									-0.99 (0.77)	0.199
**Hospital level**										
Regional hospitals vs. medical centers	**1.82** (**0.61)**	**0.003**	0.95 (0.59)	0.104	1.16 (0.75)	0.121	0.56 (0.71)	0.426	**1.47 (0.58)**	**0.012**
District hospitals vs. medical centers	0.92 (0.60)	0.123	**1.25 (0.58)**	**0.030**	0.52 (0.73)	0.474	0.01 (0.70)	0.995	0.89 (0.57)	0.119
Clinic vs. medical centers	1.15 (0.57)	0.043	**1.75 (0.55)**	**0.002**	0.78 (0.69)	0.264	0.60 (0.66)	0.363	**1.17 (0.54)**	**0.030**
Age	**0.03** (**0.01)**	**0.017**			**0.05** (**0.01)**	**<0.001**			**0.04** (**0.01)**	**0.002**
**Education level**										
Junior high school vs. less than elementary school	0.59 (0.36)	0.099							0.47 (0.34)	0.166
Senior high school vs. less than elementary school	0.36 (0.35)	0.301							0.36 (0.33)	0.281
College or above vs. less than elementary school	**0.84** (**0.37)**	**0.023**							**0.94** (**0.36)**	**0.009**
**Employment status**										
Employed vs. unemployed			**0.93** (**0.28)**	**0.001**	**0.92** (**0.37)**	**0.013**				
**Marital status**										
Married/living with someone vs. other (single, widowed, divorced)			**0.95** (**0.26)**	**<0.001**			**2.70** (**0.31)**	**<0.001**	**0.83** (**0.26)**	**0.001**
**Karnofsky Performance Scale**	**0.13** (**0.01)**	**<0.001**	**0.06** (**0.01)**	**<0.001**	**0.11** (**0.01)**	**<0.001**	**0.06** (**0.01)**	**<0.001**	**0.10** (**0.01)**	**<0.001**
**Dialysis duration (year)**	**-0.06 (0.03)**	**0.038**	**-0.06 (0.03)**	**0.043**	**-0.08 (0.03)**	**0.030**				
**Dialysis modality**^**a**^ **×dialysis duration**	0.13 (0.07)	0.063	**0.18 (0.07)**	**0.005**	**0.26 (0.08)**	**0.002**				
**Dialysis modality ×hospital level 4**^**b**^	**-5.52** (**1.77)**	**0.002**	**-5.06** (**1.72)**	**0.003**	**-5.01** (**2.17)**	**0.021**	-3.93 (2.08)	0.059	**-5.34** (**1.69)**	**0.002**
**Dialysis modality ×hospital level 3**^**b**^	-1.16 (0.97)	0.233	**-2.33** (**0.95)**	**0.014**	-1.88 (1.19)	0.116	-0.40 (1.14)	0.723	-1.66 (0.92)	0.072
**Dialysis modality ×hospital level 2**^**b**^	**-2.97** (**0.73)**	**<0.001**	**-3.75** (**0.71)**	**<0.001**	**-3.82** (**0.90)**	**<0.001**	**-3.07** (**0.85)**	**<0.001**	**-3.59** (**0.69)**	**<0.001**

^a^ Dialysis modality: 1 = peritoneal dialysis; 0 = hemodialysis.

^b^ Hospital levels: 1 = medical centers; 2 = regional hospitals; 3 = district hospitals; 4 = clinics.

Abbreviations: B, unstandardized coefficients; SE, standard error; HD, hemodialysis; PD, peritoneal dialysis

## Discussion

QOL is considered an important treatment goal. To the best of our knowledge, our study is the first national survey to compare the QOL between Taiwanese HD and PD patients. According to the present findings, PD patients scored higher in total QOL and all four subscales of the QLI. After adjusting for confounding variables, the QOL of PD patients who visited medical centers was significantly higher than that of HD patients. In contrast, the QOL of PD patients who visited clinics was lower than that of HD patients.

There is no copayment for PD or HD treatments in Taiwan’s National Health Insurance. ESRD patients can decide on the modality of dialysis therapy according to their personal preference; therefore, the assignment of PD and HD could not be randomized because 95% of the patients have their own preference for dialysis modality when they are randomized to the centers and will withdraw from the study [14]. Because the assignment of PD and HD could not be randomized, the characteristics of the two groups were significantly different. PD patients were younger, better educated, more likely to be employed, and had lower hemoglobin and albumin concentrations than HD patients. Similar discrepancies were also found in previous studies [6,11–13,17]. However, in our study, the diabetic nephropathy rate was not different between PD and HD patients, which was not consistent with the results of some previous studies [12,13]. The possible reason might be that with the advancement of medical improvements and the usage of low-glucose degradation product (GDP) or icodextrin dialysis fluid, PD treatment leads to better improvements in the QOL of diabetic ESRD patients. This enables diabetic patients to not worry about possible cardiovascular complications due to PD and causes them to opt for HD instead, and they can choose their preferred modality.

After adjusting for confounding factors, with regard to visits to medical centers, the QOL of PD patients was 2.81 significantly higher than that of HD patients. With regard to clinic visits, the QOL of PD patients was 2.53 lower than that of HD patients. This result conflicts with the results of studies by Mau et al. [12] in Southern Taiwan and Peng et al. [13] in Northern Taiwan.

Mau et al. [12] revealed that while controlling for patient characteristics, comorbid conditions, and laboratory values, PD patients had higher bodily pain scores than HD patients, while HD patients had higher social functioning scores than PD patients (p<0.05). Such discrepancies may be attributed to patient selection bias because in that study, HD patients were enrolled in two hospital-based dialysis units in southern Taiwan, and the average age was 53 years; however, the average age of the Taiwanese HD population was approximately 60 years. Because our study enrolled a much larger group of patients from Taiwan, there was likely less patient selection bias.

The results of the study by Peng et al. [13] showed that after adjusting for age, diabetes, cardiovascular disease history, dialysis duration, and levels of albumin and hematocrit, PD patients tended to have higher scores for bodily pain (p = 0.014), vitality (p = 0.017), and social function (p = 0.009). The possible reason for this might also be selection bias because a study mentioned that patients belonging to the following categories tend to choose PD: younger age, female sex, married or living with someone, higher education level, and employed [18]. However, these confounding factors were not controlled for during regression, which may affect the comparison of QOL between PD and HD patients.

The aforementioned studies did not consider the effects of regions and hospital levels. In our study, the distribution rate of PD and HD in different regions and hospital levels was used for equal sampling. After controlling for demographic, environmental, and disease characteristics, we found interactions between the hospital levels and dialysis modality. In medical centers, the QOL of patients undergoing PD was higher than that of those undergoing HD, but this was reversed in clinics. One possible reason is that medical centers have an interdisciplinary team for PD care. In contrast, regional hospitals and lower levels may have insufficient manpower resources, which affects clinical care. In Taiwan, clinics are mostly dialysis centers that focus on HD care, and only a few PD patients visit clinics. Therefore, the care of PD patients is performed by HD nurses, which may provide insufficient support for PD patients, including PD management, follow-up, and ongoing support by renal health care professionals. However, as our study did not examine the quality of PD care teams at different hospital levels, this possibility should be further examined in the future. Another reason may be the degree of illness acceptance. Jankowska-Polanska et al. [19] proposed the concept of illness acceptance: the higher the degree of illness acceptance, the greater the QOL. Although their study mainly focused on HD patients, we can further infer that patients with high degrees of illness acceptance will actively seek quality medical care and cooperate with medical instructions or health education.

This study had several limitations. First, it was a cross-sectional observational study. We did not exclude differences in QOL before dialysis. Although randomized controlled trials are considered the gold standard to estimate the effects of treatments and interventions on outcomes, HD and PD differ profoundly, and randomized comparisons have proven impossible [14]. Observational studies remain the major methods for comparing outcomes between PD and HD patients. Second, this study included all prevalent ESRD patients. Time-dependent QOL changes in HD and PD patients could not be investigated in this study. Third, further longitudinal comparison studies using an incident dialysis cohort are needed. These limitations mean that our results should be interpreted with caution.

In conclusion, the present study showed that Taiwanese PD patients who visited medical centers had better QOL than HD patients. Our findings improve our understanding of QOL in patients treated with different dialysis modalities in Taiwan.

## Supporting information

S1 Table Comparison of quality of life index scores between CAPD and APD patients. (DOCX)

S1 File Quality of life index questionnaire (English). (PDF)

S2 File Quality of life index questionnaire (Chinese). (PDF)

S3 File QOL data. (SAV)
