# Supplementary material for: A comparison of quality of life between patients treated with different dialysis modalities in Taiwan
Source: PLoS One. 2020 Jan 6;15(1):e0227297. doi: 10.1371/journal.pone.0227297 (PMC6944387; doi:10.1371/journal.pone.0227297)
Supplement: S1 Table — (DOCX) [file pone.0227297.s001.docx]

**Supplementary material**

**Table S1. Comparison of Quality of Life Index Scores between CAPD and APD Patients.**

| Subscale score (0–30) | CAPD (n=224)  Mean±SD | APD (n=163)  Mean±SD | p-value^a^ |
| --- | --- | --- | --- |
| Total quality of life | 20.28±4.12 | 19.49±4.14 | 0.066 |

^a^ Student’s t-test. CAPD, continuous ambulatory peritoneal dialysis; APD, automated peritoneal dialysis; SD, standard deviation.
