# Supplementary material for: A comparison of quality of life between patients treated with different dialysis modalities in Taiwan
Source: PLoS One. 2020 Jan 6;15(1):e0227297. doi: 10.1371/journal.pone.0227297 (PMC6944387; doi:10.1371/journal.pone.0227297)
Supplement: S1 File — (PDF) [file pone.0227297.s002.pdf]

Ferrans and Powers
QUALITY OF LIFE INDEX©
DIALYSIS VERSION - III

PART 1. For each of the following, please choose the answer that best describes how ***satisfied*** you are with that area of your life. Please mark your answer by circling the number. There are no right or wrong answers.

**HOW *SATISFIED* ARE YOU WITH:**	Very Dissatisfied	Moderately Dissatisfied	Slightly Dissatisfied	Slightly Satisfied	Moderately Satisfied	Very Satisfied
1. Your health?	1	2	3	4	5	6
2. Your health care?	1	2	3	4	5	6
3. The amount of energy you have for everyday activities?	1	2	3	4	5	6
4. Your ability to take care of yourself without help?	1	2	3	4	5	6
5. The likelihood you will get a kidney transplant?	1	2	3	4	5	6
6. The changes you have had to make in your life because of kidney failure (such as diet and need for dialysis)?	1	2	3	4	5	6
7. The amount of control you have over your life?	1	2	3	4	5	6
8. Your chances of living as long as you would like?	1	2	3	4	5	6
9. Your family's health?	1	2	3	4	5	6
10. Your children?	1	2	3	4	5	6
11. Your family's happiness?	1	2	3	4	5	6
12. Your sex life?	1	2	3	4	5	6
13. Your spouse, lover, or partner?	1	2	3	4	5	6
14. Your friends?	1	2	3	4	5	6
15. The emotional support you get from your family?	1	2	3	4	5	6

(Please Go To Next Page)

© Copyright 1984 & 1998 Carol Estwing Ferrans and Marjorie J. Powers (Do not use without permission).

HOW *SATISFIED* ARE YOU WITH:	Very Dissatisfied	Moderately Dissatisfied	Slightly Dissatisfied	Slightly Satisfied	Moderately Satisfied	Very Satisfied
16. The emotional support you get from people other than your family?	1	2	3	4	5	6
17. Your ability to take care of family responsibilities?	1	2	3	4	5	6
18. How useful you are to others?	1	2	3	4	5	6
19. The amount of worries in your life?	1	2	3	4	5	6
20. Your neighborhood?	1	2	3	4	5	6
21. Your home, apartment, or place where you live?	1	2	3	4	5	6
22. Your job (if employed)?	1	2	3	4	5	6
23. Not having a job (if unemployed, retired, or disabled)?	1	2	3	4	5	6
24. Your education?	1	2	3	4	5	6
25. How well you can take care of your financial needs?	1	2	3	4	5	6
26. The things you do for fun?	1	2	3	4	5	6
27. Your chances for a happy future?	1	2	3	4	5	6
28. Your peace of mind?	1	2	3	4	5	6
29. Your faith in God?	1	2	3	4	5	6
30. Your achievement of personal goals?	1	2	3	4	5	6
31. Your happiness in general?	1	2	3	4	5	6
32. Your life in general?	1	2	3	4	5	6
33. Your personal appearance?	1	2	3	4	5	6
34. Yourself in general?	1	2	3	4	5	6

(Please Go To Next Page)

© Copyright 1984 &1998 Carol Estwing Ferrans and Marjorie J. Powers (Do not use without permission).

PART 2. For each of the following, please choose the answer that best describes how ***important*** that area of your life is to you. Please mark your answer by circling the number. There are no right or wrong answers.

**HOW *IMPORTANT* TO YOU IS:**	Very Unimportant	Moderately Unimportant	Slightly Unimportant	Slightly Important	Moderately Important	Very Important
1. Your health?	1	2	3	4	5	6
2. Your health care?	1	2	3	4	5	6
3. Having enough energy for everyday activities?	1	2	3	4	5	6
4. Taking care of yourself without help?	1	2	3	4	5	6
5. Getting a kidney transplant?	1	2	3	4	5	6
6. The changes you have had to make in your life because of kidney failure (such as diet and need for dialysis)?	1	2	3	4	5	6
7. Having control over your life?	1	2	3	4	5	6
8. Living as long as you would like?	1	2	3	4	5	6
9. Your family's health?	1	2	3	4	5	6
10. Your children?	1	2	3	4	5	6
11. Your family's happiness?		2	3	4	5	6
12. Your sex life?	1	2	3	4	5	6
13. Your spouse, lover, or partner?	1	2	3	4	5	6
14. Your friends?	1	2	3	4	5	6
15. The emotional support you get from your family?	1	2	3	4	5	6
16. The emotional support you get from people other than your family?	1	2	3	4	5	6

(Please Go To Next Page)

© Copyright 1984 & 1998 Carol Estwing Ferrans and Marjorie J. Powers (Do not use without permission).

HOW *IMPORTANT* TO YOU IS:	Very Unimportant	Moderately Unimportant	Slightly Unimportant	Slightly Important	Moderately Important	Very Important
17. Taking care of family responsibilities?	1	2	3	4	5	6
18. Being useful to others?	1	2	3	4	5	6
19. Having no worries?	1	2	3	4	5	6
20. Your neighborhood?	1	2	3	4	5	6
21. Your home, apartment, or place where you live?	1	2	3	4	5	6
22. Your job (if employed)?	1	2	3	4	5	6
23. Having a job (if unemployed, retired, or disabled)?	1	2	3	4	5	6
24. Your education?	1	2	3	4	5	6
25. Being able to take care of your financial needs?	1	2	3	4	5	6
26. Doing things for fun?	1	2	3	4	5	6
27. Having a happy future?	1	2	3	4	5	6
28. Peace of mind?	1	2	3	4	5	6
29. Your faith in God?	1	2	3	4	5	6
30. Achieving your personal goals?	1	2	3	4	5	6
31. Your happiness in general?	1	2	3	4	5	6
32. Being satisfied with life?	1	2	3	4	5	6
33. Your personal appearance?	1	2	3	4	5	6
34. Are you to yourself?	1	2	3	4	5	6

© Copyright 1984 & 1998 Carol Estwing Ferrans and Marjorie J. Powers (Do not use without permission).
