# Supplementary material for: A comparison of quality of life between patients treated with different dialysis modalities in Taiwan
Source: PLoS One. 2020 Jan 6;15(1):e0227297. doi: 10.1371/journal.pone.0227297 (PMC6944387; doi:10.1371/journal.pone.0227297)
Supplement: S2 File — (PDF) [file pone.0227297.s003.pdf]

健康相關生活品質問卷

第一部份 生活品質指標：滿意度 編號：______

◎填寫說明：每個人對下列項目生活的滿意程度不同。所有的答案並無”對”或”錯”。請針對下列項目圈選您認為最近**兩個星期**最適合描述您自己各方面生活的**滿意程度。**

就您目前下列各項的生活現況，您的**滿意度**為何？	非常不滿意	中等程度的不滿意	稍微不滿意	稍微滿意	中等程度的滿意	非常滿意
1.健康						
2.所接受的整體醫療照顧						
3.應付日常生活的體力						
4.有能力照顧自己，不需要別人協助						
5.對得到腎臟移植的希望						
6.因腎衰竭所做的生活改變（如飲食及需要透析）						
7.掌控自己生活的程度						
8.如自己所願，活下來的機會						
9.家人的健康						
10.子女						

就您目前下列各項的生活現況，您的**滿意度**為何？	非常不滿意	中等程度的不滿意	稍微不滿意	稍微滿意	中等程度的滿意	非常滿意
11. 家庭的快樂幸福						
12. 性生活						
13. 配偶、愛人或親密伴侶						
14. 朋友						
15. 來自家人的情緒支持						
16. 來自家人以外的情緒支持						
17. 負擔家庭責任的能力						
18. 幫助別人的能力						
19. 生活中的憂慮程度						
20. 您的左鄰右舍						
21. 所居住的房子（或公寓）及地點						
22. 工作（若您就業中請填此題）						

就您目前下列各項的生活現況，您的**滿意度**為何？	非常不滿意	中等程度的不滿意	稍微不滿意	稍微滿意	中等程度的滿意	非常滿意
23.沒有工作（若您無工作請填此題）						
24.教育程度						
25.能滿足目前經濟狀況的程度						
26.做您覺得有趣的事						
27.擁有美好快樂的未來						
28.平靜的心						
29.宗教信仰						
30.達到個人的目標						
31.整體生活的快樂程度						
32.整體而言，對自己生活的感受						
33.對自己外表的感受						
34.整體而言，對自己的滿意度						

<u>第二部份 綜合自我評估</u>

◎填寫說明：請依您**<u>最近兩個星期</u>**的情況，回答下列題目；「0」端代表生活品質**<u>最差</u>**的狀態，「100」端代表生活品質**<u>最佳</u>**的狀態，根據此觀點，請在下列的長條圖中，以**<u>箭頭及數字</u>**的方式，標出您的情況，謝謝。

例如：　整體而言，我對自己<u>健康相關生活品質</u>的滿意程度

65

最差　　　　　　　　　　　　　　　　　　　　　　　　　　　　　　最佳

0　10　20　30　40　50　60　70　80　90　100

綜合而言，我在**洗腎之後**，我對自己<u>健康相關生活品質</u>的滿意程度

最差　　　　　　　　　　　　　　　　　　　　　　　　　　　　　　最佳

0　10　20　30　40　50　60　70　80　90　100

第三部份 生活品質指標：重要度

◎填寫說明：每個人對下列項目生活的滿意程度不同。所有的答案並無”對”或”錯”。請針對下列項目圈選您認為**最近兩個星期**最適合描述您自己各方面生活的**重要程度。**

下列項目對您的生活有**多重要**？	非常不重要	中等程度的不重要	稍微不重要	稍微重要	中等程度的重要	非常重要
1.健康						
2.所接受的整體醫療照顧						
3.應付日常生活的體力						
4.有能力照顧自己，不需要別人協助						
5.得到腎臟移植						
6.因腎衰竭所做的生活改變（如飲食及需要透析）						
7.可以掌控自己生活的程度						
8.如自己所願，活下來的機會						
9.家人的健康						
10.子女						

下列項目對您的生活有**多重要**？	非常不重要	中等程度的不重要	稍微不重要	稍微重要	中等程度的重要	非常重要
11. 家庭的快樂幸福						
12. 性生活						
13. 配偶、愛人或親密伴侶						
14. 朋友						
15. 來自家人的情緒支持						
16. 來自家人以外的情緒支持						
17. 負擔家庭責任的能力						
18. 幫助別人的能力						
19. 生活中沒有憂慮						
20. 您的左鄰右舍						
21. 所居住的房子（或公寓）及地點						
22. 工作（若您就業中請填此題）						

下列項目對您的生活有**多重要**？	非常不重要	中等程度的不重要	稍微不重要	稍微重要	中等程度的重要	非常重要
23.找到工作（若您無工作、退休或殘障請填此題）						
24.教育程度						
25.有能力滿足自己的經濟需求						
26.做自己有興趣的事						
27.擁有美好快樂的未來						
28.平靜的心						
29.宗教信仰						
30.達到個人的目標						
31.整體生活的快樂程度						
32.滿意自己的生活						
33.對自己外表的感受						
34.能面對自己						
